# Supplementary material for: Prenatal care coverage and correlates of HIV testing in sub-Saharan Africa: Insight from demographic and health surveys of 16 countries
Source: PLoS One. 2020 Nov 9;15(11):e0242001. doi: 10.1371/journal.pone.0242001 (PMC7652338; doi:10.1371/journal.pone.0242001)
Supplement: S1 Table — (DOCX) [file pone.0242001.s001.docx]

Table S1: Adjusted and unadjusted logistic regression models showing factors associated with prenatal uptake of HIV testing in Angola, Cameroun and Chad

| Variables | Angola | | Cameroon | | Chad | |
| --- | --- | --- | --- | --- | --- | --- |
| Knowledge of MTCT | UOR [95% CI] | AOR [95% CI] | UOR [95% CI] | AOR [95% CI] | UOR [95% CI] | AOR [95% CI] |
| Low | Ref | Ref | Ref | Ref | Ref | Ref |
| Moderate | 11.35 [9.34,13.80]^***^ | 5.75 [4.63,7.15]*** | 4.90 [3.80,6.33]^***^ | 3.44 [2.58,4.60]*** | 35.56 [23.86,52.99]^***^ | 31.53 [20.88,47.60]*** |
| High | 18.13 [15.55,21.14]^***^ | 9.41 [7.96,11.12]*** | 7.22 [5.77,9.04]^***^ | 4.94 [3.83,6.38]*** | 46.76 [32.67,66.92]^***^ | 43.39 [29.97,62.82]*** |
| Age group in years |  |  |  |  |  |  |
| 15-19 | Ref | Ref | Ref | Ref | Ref | Ref |
| 20-24 | 1.24 [1.05,1.46]^*^ | 1.27 [1.02,1.57]* | 1.47 [1.15,1.88]^**^ | 1. m6 | 1.07 [0.74,1.56] | 0.79 [0.51,1.22] |
| 25-34 | 1.24 [1.06,1.45]^**^ | 1.53 [1.23,1.91]*** | 1.28 [1.03,1.60]^*^ | 1.28 [0.98,1.69] | 1.03 [0.69,1.54] | 1.07 [0.72,1.60] |
| 35-49 | 1.00 [0.83,1.21] | 1.64 [1.26,2.14]*** | 1.06 [0.81,1.39] | 1.26 [0.90,1.76] | 1.03 [0.69,1.54] | 1.27 [0.79,2.04] |
| Marital Status |  |  |  |  |  |  |
| Never Married | Ref | Ref | Ref | Ref | Ref | Ref |
| Currently married | 0.70 [0.58,0.84]^***^ | 0.59 [0.45,0.77]*** | 0.34 [0.26,0.45]^***^ | 1.04 [0.73,1.46] | 0.52 [0.22,1.21] | 0.65 [0.22,1.90] |
| Previously married | 1.04 [0.83,1.30] | 1.03 [0.76,1.40] | 0.38 [0.25,0.57]^***^ | 0.8 [0.50,1.28] | 0.52 [0.31,2.11] | 0.7 [0.22,2.28] |
| Cohabiting | 1.00 [0.88,1.15] | 0.82 [0.68,0.99]* | 0.69 [0.50,0.95]^*^ | 1.03 [0.71,1.49] | 0.83 [0.33,2.07] | 0.78 [0.25,2.41] |
| Education level |  |  |  |  |  |  |
| None | Ref | Ref | Ref | Ref | Ref | Ref |
| Primary | 3.15 [2.74,3.62]^***^ | 1.98 [1.67,2.34]*** | 3.46 [2.86,4.20]^***^ | 2.59 [2.08,3.22]*** | 2.68 [2.07,3.47]^***^ | 1.92 [1.42,2.60]*** |
| Secondary & Higher | 14.52 [12.32,17.10]^***^ | 3.31 [2.68,4.10]*** | 15.53 [12.31,19.60]^***^ | 6.32 [4.72,8.45] *** | 5.07 [3.84,6.70]^***^ | 2.63 [1.81,3.84]*** |
| Wealth Status |  |  |  |  |  |  |
| Poor | Ref | Ref | Ref | Ref | Ref | Ref |
| Middle | 4.84 [4.22,5.54]^***^ | 1.92 [1.59,2.33]*** | 3.16 [2.59,3.86]^***^ | 1.60 [1.24,2.07]*** | 0.85 [0.59,1.22] | 0.92 [0.62,1.37] |
| Rich | 17.89 [14.94,21.43]^***^ | 3.52 [2.72,4.55]*** | 9.74 [7.55,12.55]^***^ | 2.55 [1.77,3.69]*** | 2.19 [1.71,2.81]^***^ | 1.09 [0.78,1.54] |
| Residence |  |  |  |  |  |  |
| Rural | Ref | Ref | Ref | Ref | Ref | Ref |
| Urban | 6.18 [5.49,6.95]^***^ | 1.73 [1.45,2.05]*** | 3.40 [2.85,4.06]^***^ | 1.07 [0.83,1.38] | 3.93 [3.15,4.91]^***^ | 2.63 [1.87,3.71] *** |
| Media Exposure |  |  |  |  |  |  |
| Low | Ref | Ref | Ref | Ref | Ref | Ref |
| Moderate | 3.96 [3.50,4.48]^***^ | 1.47 [1.25,1.73]*** | 4.70 [3.91,5.66]^***^ | 1.69 [1.34,2.14]*** | 3.13 [2.45,3.98]^***^ | 1.34 [0.99,1.80] |
| High | 12.02 [9.86,14.66]^***^ | 1.64 [1.25,2.15]*** | 13.03 [8.90,19.07]^***^ | 2.70 [1.75,4.18]*** | 6.04 [4.37,8.35]^***^ | 1.86 [1.15,3.00]* |
| Health Insurance Cover |  |  |  |  |  |  |
| No | Ref | Ref | Ref | Ref | Ref | Ref |
| Yes | 1.49 [1.16,1.91]^**^ | 1.16 [0.83,1.61] | 4.30 [1.55,11.90]^**^ | 1.14 [0.38,3.48] | 98.68 [21.79,447.03]^***^ | 6.38 [1.33,30.70]* |

AOR is the adjusted odds ratio, UOR is the unadjusted odds ratio, ref is the reference; Exponentiated coefficients; 95% confidence intervals in brackets

^*^ *p* < 0.05, ^**^ *p* < 0.01, ^***^ *p* < 0.001
