# Supplementary material for: Prenatal care coverage and correlates of HIV testing in sub-Saharan Africa: Insight from demographic and health surveys of 16 countries
Source: PLoS One. 2020 Nov 9;15(11):e0242001. doi: 10.1371/journal.pone.0242001 (PMC7652338; doi:10.1371/journal.pone.0242001)
Supplement: S2 Table — (DOCX) [file pone.0242001.s002.docx]

Table S2: Adjusted and unadjusted logistic regression models showing factors associated with prenatal uptake of HIV testing in Benin, Guinea and Mali

| Variables | Benin | | Guinea | | Mali | |
| --- | --- | --- | --- | --- | --- | --- |
| Knowledge of MTCT | UOR [95% CI] | AOR [95% CI] | UOR [95% CI] | AOR [95% CI] | UOR [95% CI] | AOR [95% CI] |
| Low | Ref | Ref | Ref | Ref | Ref | Ref |
| Moderate | 37.20 [27.47,50.37]^***^ | 40.11 [29.13,55.22]*** | 3.95 [2.97,5.25]^***^ | 2.89 [2.13,3.91]*** | 4.58 [3.55,5.89]^***^ | 3.11 [2.35,4.12]*** |
| High | 33.10 [26.42,41.47]^***^ | 38.57 [30.45,48.86]*** | 5.46 [4.33,6.89]^***^ | 4.18 [3.27,5.34]*** | 2.93 [2.38,3.61]^***^ | 2.27 [1.81,2.85]*** |
| Age group in years |  |  |  |  |  |  |
| 15-19 | Ref | Ref | Ref | Ref | Ref | Ref |
| 20-24 | 1.19 [0.89,1.60] | 1.22 [0.84,1.77] | 1.01 [0.75,1.36] | 0.93 [0.67,1.29] | 1.47 [1.09,1.97]^*^ | 1.2 [0.86,1.67] |
| 25-34 | 1.41 [1.07,1.85]^*^ | 1.55 [1.08,2.22]* | 0.99 [0.76,1.29] | 0.99 [0.73,1.35] | 1.46 [1.11,1.92]^**^ | 1.57 [1.14,2.15]** |
| 35-49 | 1.49 [1.10,2.01]^**^ | 1.91 [1.28,2.85]** | 0.75 [0.55,1.01] | 0.93 [0.65,1.32] | 1.32 [0.97,1.80] | 1.60 [1.12,2.30]* |
| Marital Status |  |  |  |  |  |  |
| Never Married | Ref | Ref | Ref | Ref | Ref | Ref |
| Currently married | 0.58 [0.41,0.80]^**^ | 0.63 [0.39,1.01] | 0.63 [0.43,0.92]^*^ | 1.12 [0.72,1.76] | 0.66 [0.43,1.02] | 1.19 [0.72,1.96] |
| Previously married | 0.59 [0.34,1.01] | 0.7 [0.34,1.44] | 1.35 [0.69,2.62] | 2.19 [1.03,4.65]* | 0.64 [0.28,1.47] | 0.83 [0.33,2.07] |
| Cohabiting | 0.81 [0.57,1.15] | 0.9 [0.55,1.48] | 0.97 [0.47,2.01] | 1.11 [0.50,2.49] | 0.71 [0.22,2.29] | 0.91 [0.26,3.14] |
| Education level |  |  |  |  |  |  |
| None | Ref | Ref | Ref | Ref | Ref | Ref |
| Primary | 1.72 [1.45,2.04]^***^ | 1.28 [1.02,1.60]* | 1.95 [1.52,2.50]^***^ | 1.26 [0.95,1.67] | 2.09 [1.65,2.66]^***^ | 1.57 [1.20,2.04]*** |
| Secondary & Higher | 2.45 [2.09,2.89]^***^ | 1.71 [1.34,2.18]*** | 4.48 [3.59,5.59]^***^ | 1.61 [1.22,2.11]*** | 6.02 [4.97,7.29]^***^ | 2.53 [1.99,3.21]*** |
| Wealth Status |  |  |  |  |  |  |
| Poor | Ref | Ref | Ref | Ref | Ref | Ref |
| Middle | 1.62 [1.33,1.97]^***^ | 1.72 [1.36,2.18]*** | 1.67 [1.28,2.18]^***^ | 1.36 [1.02,1.80]* | 1.65 [1.24,2.20]^***^ | 1.42 [1.06,1.91]* |
| Rich | 2.79 [2.38,3.26]^***^ | 2.18 [1.74,2.73]*** | 1.67 [4.40,6.64]^***^ | 2.10 [1.55,2.86]*** | 7.14 [5.73,8.90]^***^ | 2.84 [2.14,3.77] *** |
| Residence |  |  |  |  |  |  |
| Rural | Ref | Ref | Ref | Ref | Ref | Ref |
| Urban | 1.73 [1.51,1.98]^***^ | 1.24 [1.03,1.50]* | 4.92 [4.11,5.89]^***^ | 2.01 [1.53,2.65]*** | 4.65 [3.92,5.50]^***^ | 1.76 [1.39,2.22]*** |
| Media Exposure |  |  |  |  |  |  |
| Low | Ref | Ref | Ref | Ref | Ref | Ref |
| Moderate | 1.77 [1.51,2.07]^***^ | 1.40 [1.15,1.70]*** | 2.23 [1.80,2.78]^***^ | 1.31 [1.03,1.66]* | 2.38 [1.81,3.13]^***^ | 1.63 [1.22,2.18]*** |
| High | 3.49 [2.86,4.25]^***^ | 2.29 [1.70,3.07] *** | 5.67 [4.31,7.46]^***^ | 1.70 [1.23,2.36]** | 5.72 [4.33,7.55]^***^ | 2.06 [1.51,2.80]*** |
| Health Insurance Cover |  |  |  |  |  |  |
| No | Ref | Ref | Ref | Ref | Ref | Ref |
| Yes | 4.20 [2.37,7.43]^***^ | 1.55 [0.67,3.62] | 8.21 [3.72,18.11]^***^ | 2.72 [1.12,6.64]* | 7.66 [5.46,10.74]^***^ | 2.47 [1.68,3.64]*** |

AOR is the adjusted odds ratio, UOR is the unadjusted odds ratio, ref is the reference; Exponentiated coefficients; 95% confidence intervals in brackets

^*^ *p* < 0.05, ^**^ *p* < 0.01, ^***^ *p* < 0.001
