# Supplementary material for: Prenatal care coverage and correlates of HIV testing in sub-Saharan Africa: Insight from demographic and health surveys of 16 countries
Source: PLoS One. 2020 Nov 9;15(11):e0242001. doi: 10.1371/journal.pone.0242001 (PMC7652338; doi:10.1371/journal.pone.0242001)
Supplement: S3 Table — (DOCX) [file pone.0242001.s003.docx]

Table S3: Adjusted and unadjusted logistic regression models showing factors associated with prenatal uptake of HIV testing in Senegal, Burundi and Ethiopia

| Variables | Senegal | | Burundi | | Ethiopia | |
| --- | --- | --- | --- | --- | --- | --- |
| Knowledge of MTCT | UOR [95% CI] | AOR [95% CI] | UOR [95% CI] | AOR [95% CI] | UOR [95% CI] | AOR [95% CI] |
| Low | Ref | Ref | Ref | Ref | Ref | Ref |
| Moderate | 2.23 [1.84,2.69]^***^ | 1.98 [1.62,2.42]*** | 36.73 [24.77,54.45]^***^ | 34.98 [23.24,52.63]*** | 5.79 [4.62,7.24]^***^ | 3.68 [2.86,4.73]*** |
| High | 2.51 [2.18,2.90]^***^ | 2.56 [2.20,2.98]*** | 47.90 [33.80,67.88]^***^ | 47.90 [33.30,68.90]*** | 6.34 [5.21,7.72]^***^ | 4.21 [3.38,5.24]*** |
| Age group in years |  |  |  |  |  |  |
| 15-19 | Ref | Ref | Ref | Ref | Ref | Ref |
| 20-24 | 1.52 [1.21,1.91]^***^ | 1.37 [1.08,1.76]* | 1.93 [1.23,3.04]^**^ | 1.85 [1.06,3.24]* | 1.14 [0.87,1.50] | 0.99 [0.71,1.37] |
| 25-34 | 1.72 [1.39,2.13]^***^ | 1.57 [1.24,1.98]*** | 1.83 [1.19,2.79]^**^ | 1.29 [0.75,2.22] | 1.05 [0.81,1.37] | 1.05 [0.76,1.45] |
| 35-49 | 1.88 [1.49,2.39]^***^ | 1.92 [1.48,2.49]*** | 1.57 [1.01,2.45]^*^ | 1.06 [0.60,1.88] | 0.74 [0.56,0.99]^*^ | 1.05 [0.73,1.50] |
| Marital Status |  |  |  |  |  |  |
| Never Married | Ref | Ref | Ref | Ref | Ref | Ref |
| Currently married | 1.12 [0.81,1.56] | 1.37 [0.95,1.98] | 1.66 [1.07,2.57]^*^ | 1.96 [1.11,3.44]* | 0.38 [0.18,0.80]^*^ | 1.01 [0.39,2.63] |
| Previously married | 0.95 [0.58,1.57] | 0.92 [0.54,1.59] | 1.38 [0.76,2.50] | 1.51 [0.75,3.07] | 0.45 [0.20,1.01] | 1.11 [0.39,3.10] |
| Cohabiting | 2.98 [0.32,27.27] | 5.9 [0.59,59.39] | 1.04 [0.66,1.64] | 1.32 [0.75,2.33] | 0.80 [0.31,2.10] | 1.68 [0.51,5.54] |
| Education level |  |  |  |  |  |  |
| None | Ref | Ref | Ref | Ref | Ref | Ref |
| Primary | 1.62 [1.38,1.90]^***^ | 1.34 [1.13,1.60]*** | 1.19 [0.98,1.46] | 1.16 [0.91,1.47] | 3.37 [2.89,3.92]^***^ | 2.09 [1.73,2.51]*** |
| Secondary & Higher | 2.38 [1.97,2.86]^***^ | 1.85 [1.49,2.30]*** | 2.24 [1.57,3.17]^***^ | 1.29 [0.83,2.01] | 14.53 [11.27,18.75]^***^ | 4.25 [3.12,5.78]*** |
| Wealth Status |  |  |  |  |  |  |
| Poor | Ref | Ref | Ref | Ref | Ref | Ref |
| Middle | 1.81 [1.55,2.12]^***^ | 1.38 [1.15,1.65]*** | 1.35 [1.04,1.76]^*^ | 0.96 [0.71,1.31] | 2.09 [1.70,2.57]^***^ | 1.53 [1.22,1.91]*** |
| Rich | 3.56 [2.98,4.26]^***^ | 2.25 [1.78,2.84]*** | 1.66 [1.33,2.06]^***^ | 0.74 [0.55,0.99]* | 8.15 [6.95,9.56]^***^ | 2.47 [2.00,3.05]*** |
| Residence |  |  |  |  |  |  |
| Rural | Ref | Ref | Ref | Ref | Ref | Ref |
| Urban | 2.26 [1.96,2.60]^***^ | 1.37 [1.15,1.64]*** | 1.99 [1.45,2.74]^***^ | 1.70 [1.14,2.53]** | 9.42 [7.76,11.44]^***^ | 2.86 [2.21,3.71]*** |
| Media Exposure |  |  |  |  |  |  |
| Low | Ref | Ref | Ref | Ref | Ref | Ref |
| Moderate | 2.49 [2.03,3.06]^***^ | 1.67 [1.35,2.08]*** | 1.76 [1.44,2.16]^***^ | 1.55 [1.22,1.98]*** | 3.80 [3.27,4.42]^***^ | 1.44 [1.19,1.73]*** |
| High | 4.29 [3.44,5.37]^***^ | 1.89 [1.47,2.43]*** | 3.02 [1.53,5.96]^**^ | 1.75 [0.81,3.79] | 17.42 [12.02,25.25]^***^ | 2.01 [1.29,3.13]** |
| Health Insurance Cover |  |  |  |  |  |  |
| No | Ref | Ref | Ref | Ref | Ref | Ref |
| Yes |  |  | 1.40 [1.11,1.77]^**^ | 0.97 [0.73,1.28] | 4.00 [2.69,5.94]^***^ | 3.29 [2.09,5.20]*** |

AOR is the adjusted odds ratio, UOR is the unadjusted odds ratio, ref is the reference; Exponentiated coefficients; 95% confidence intervals in brackets

^*^ *p* < 0.05, ^**^ *p* < 0.01, ^***^ *p* < 0.001
