# Supplementary material for: Prenatal care coverage and correlates of HIV testing in sub-Saharan Africa: Insight from demographic and health surveys of 16 countries
Source: PLoS One. 2020 Nov 9;15(11):e0242001. doi: 10.1371/journal.pone.0242001 (PMC7652338; doi:10.1371/journal.pone.0242001)
Supplement: S4 Table — (DOCX) [file pone.0242001.s004.docx]

Table S4: Adjusted and unadjusted logistic regression models showing factors associated with prenatal uptake of HIV testing in Rwanda, Uganda and South Africa

| Variables | Rwanda | | Uganda | | South Africa | |
| --- | --- | --- | --- | --- | --- | --- |
| Knowledge of MTCT | UOR [95% CI] | AOR [95% CI] | UOR [95% CI] | AOR [95% CI] | UOR [95% CI] | AOR [95% CI] |
| Low | Ref | Ref | Ref | Ref | Ref | Ref |
| Moderate | 9.74 [2.62,36.19]^***^ | 9.75 [2.44,38.94]** | 5.25 [3.43,8.04]^***^ | 4.23 [2.74,6.53]*** | 7.93 [4.04,15.57]^***^ | 8.49 [4.25,16.98]*** |
| High | 9.45 [2.66,33.59]^***^ | 9.89 [2.58,37.83]*** | 4.33 [2.89,6.50]^***^ | 3.42 [2.26,5.17]*** | 7.21 [4.68,11.10]^***^ | 7.86 [4.97,12.43]*** |
| Age group in years |  |  |  |  |  |  |
| 15-19 | Ref | Ref | Ref | Ref | Ref | Ref |
| 20-24 | 1.06 [0.23,4.92] | 0.98 [0.21,4.62] | 1.46 [1.07,2.00]^*^ | 1.23 [0.88,1.71] | 0.73 [0.34,1.54] | 0.61 [0.28,1.34] |
| 25-34 | 0.79 [0.19,3.34] | 0.63 [0.14,2.87] | 1.62 [0.71,1.39]^**^ | 1.37 [0.99,1.90] | 0.67 [0.34,1.35] | 0.52 [0.25,1.11] |
| 35-49 | 0.60 [0.14,2.66] | 0.51 [0.11,2.45] | 0.99 [0.71,1.39] | 0.98 [0.68,1.42] | 0.51 [0.24,1.10] | 0.40 [0.17,0.93] * |
| Marital Status |  |  |  |  |  |  |
| Never Married | Ref | Ref | Ref | Ref | Ref | Ref |
| Currently married | 1.73 [0.72,4.15] | 2.3 [0.88,6.01] | 1.11 [0.73,1.68] | 1.32 [0.83,2.08] | 1.11 [0.69,1.78] | 1.59 [0.92,2.73] |
| Previously married | 0.46 [0.17,1.24] | 0.68 [0.24,1.93] | 1.02 [0.62,1.69] | 1.23 [0.72,2.09] | 1.36 [0.41,4.52] | 1.73 [0.48,6.25] |
| Cohabiting | 0.86 [0.36,2.01] | 1.13 [0.46,2.79] | 1.11 [0.73,1.68] | 1.23 [0.79,1.91] | 1.06 [0.62,1.80] | 1.13 [0.63,2.03] |
| Education level |  |  |  |  |  |  |
| None | Ref | Ref | Ref | Ref | Ref | Ref |
| Primary | 3.13 [1.76,5.54]^***^ | 2.50 [1.36,4.61]** | 1.20 [0.91,1.57] | 1.09 [0.82,1.45] | 0.40 [0.05,3.24] | 0.3 [0.03,2.72] |
| Secondary & Higher | 4.16 [1.65,10.52]^**^ | 2.15 [0.73,6.31] | 4.47 [2.97,6.73]^***^ | 2.79 [1.78,4.37]*** | 0.65 [0.09,4.95] | 0.42 [0.05,3.64] |
| Wealth Status |  |  |  |  |  |  |
| Poor | Ref | Ref | Ref | Ref | Ref | Ref |
| Middle | 2.56 [1.08,6.11]^*^ | 1.83 [0.75,4.45] | 1.33 [1.02,1.73]^*^ | 1.13 [0.86,1.48] | 1.43 [0.85,2.38] | 1.42 [0.79,2.55] |
| Rich | 2.03 [1.12,3.66]^*^ | 1.03 [0.49,2.14] | 2.26 [1.75,2.93]^***^ | 1.14 [0.84,1.53] | 1.18 [0.76,1.83] | 1.17 [0.63,2.17] |
| Residence |  |  |  |  |  |  |
| Rural | Ref | Ref | Ref | Ref | Ref | Ref |
| Urban | 1.49 [0.75,2.96] | 1.29 [0.58,2.83] | 3.28 [2.22,4.84]^***^ | 2.14 [1.41,3.23]*** | 0.87 [0.59,1.26] | 0.58 [0.35,0.93] * |
| Media Exposure |  |  |  |  |  |  |
| Low | Ref | Ref | Ref | Ref | Ref | Ref |
| Moderate | 2.10 1.18,3.73]^*^ | 1.53 [0.83,2.81] | 1.69 [1.36,2.09]^***^ | 1.43 [1.14,1.78] ** | 2.01 [1.15,3.51]^*^ | 1.82 [0.98,3.38] |
| High | 4.81 [1.77,13.06]^**^ | 2.7 [0.86,8.42] | 3.75 [2.50,5.62]^***^ | 1.73 [1.10,2.72]* | 1.91 [1.15,3.17]^*^ | 1.53 [0.82,2.84] |
| Health Insurance Cover |  |  |  |  |  |  |
| No | Ref | Ref | Ref | Ref | Ref | Ref |
| Yes |  |  | 2.50 [0.61,10.27] | 1.68 [0.40,7.11] | 0.55 [0.26,1.15] | 0.66 [0.29,1.52] |

AOR is the adjusted odds ratio, UOR is the unadjusted odds ratio, ref is the reference; Exponentiated coefficients; 95% confidence intervals in brackets

^*^ *p* < 0.05, ^**^ *p* < 0.01, ^***^ *p* < 0.001
