# Supplementary material for: Prenatal care coverage and correlates of HIV testing in sub-Saharan Africa: Insight from demographic and health surveys of 16 countries
Source: PLoS One. 2020 Nov 9;15(11):e0242001. doi: 10.1371/journal.pone.0242001 (PMC7652338; doi:10.1371/journal.pone.0242001)
Supplement: S5 Table — (DOCX) [file pone.0242001.s005.docx]

Table S5: Adjusted and unadjusted logistic regression models showing factors associated with prenatal uptake of HIV testing in Zambia, Zimbabwe, Malawi and

| Variables | Zambia | | Zimbabwe | | Malawi | |
| --- | --- | --- | --- | --- | --- | --- |
| Knowledge of MTCT | UOR [95% CI] | AOR [95% CI] | UOR [95% CI] | AOR [95% CI] | UOR [95% CI] | AOR [95% CI] |
| Low | Ref | Ref | Ref | Ref | Ref | Ref |
| Moderate | 23.15 [15.30,35.02]^***^ | 19.44 [12.67,29.82]*** | 9.46 [5.28,16.96]^***^ | 8.92 [4.81,16.57]*** | 5.40 [4.07,7.14]^***^ | 5.31 [3.99,7.06] *** |
| High | 21.06 [15.27,29.03]^***^ | 17.12 [12.20,24.02]*** | 19.23 [11.13,33.24]^***^ | 18.54 [10.31,33.37]*** | 6.35 [5.08,7.95]^***^ | 6.00 [4.77,7.54]*** |
| Age group in years |  |  |  |  |  |  |
| 15-19 | Ref | Ref | Ref | Ref | Ref | Ref |
| 20-24 | 1.79 [1.21,2.64]^**^ | 1.46 [0.91,2.34] | 0.92 [0.54,1.55] | 0.8 [0.44,1.43] | 1.89 [1.49,2.40]^***^ | 1.59 [1.23,2.06]*** |
| 25-34 | 1.47 [1.03,2.09]^*^ | 1.34 [0.85,2.12] | 0.75 [0.46,1.21] | 0.53 [0.30,0.92]* | 1.97 [1.56,2.49]^***^ | 1.55 [1.20,2.01]*** |
| 35-49 | 1.39 [0.92,2.11] | 1.6 [0.95,2.70] | 0.70 [0.39,1.23] | 0.54 [0.28,1.03] | 1.38 [1.05,1.80]^*^ | 1.23 [0.91,1.67] |
| Marital Status |  |  |  |  |  |  |
| Never Married | Ref | Ref | Ref | Ref | Ref | Ref |
| Currently married | 0.73 [0.49,1.10] | 0.71 [0.42,1.18] | 0.37 [0.16,0.84]^*^ | 0.40 [0.16,0.98]* | 2.32 [1.74,3.09]^***^ | 2.04 [1.47,2.84]*** |
| Previously married | 0.51 [0.30,0.87]^*^ | 0.58 [0.30,1.12] | 0.34 [0.13,0.88]^*^ | 0.35 [0.13,0.96]* | 1.49 [1.05,2.11]^*^ | 1.29 [0.87,1.90] |
| Cohabiting | 0.67 [0.08,5.32] | 0.93 [0.09,9.85] | 0.56 [0.18,1.79] | 0.63 [0.18,2.17] | 2.16 [1.38,3.37]^***^ | 1.95 [1.20,3.15]** |
| Education level |  |  |  |  |  |  |
| None | Ref | Ref | Ref | Ref | Ref | Ref |
| Primary | 3.21 [2.35,4.39]^***^ | 2.91 [2.01,4.21]*** | 0.81 [0.24,2.75] | 0.62 [0.16,2.36] | 1.20 [0.94,1.53] | 1.06 [0.81,1.39] |
| Secondary & Higher | 9.46 [6.22,14.39]^***^ | 5.98 [3.53,10.14]*** | 2.11 [0.62,7.21] | 1.2 [0.31,4.67] | 1.87 [1.38,2.54]^***^ | 1.38 [0.96,1.97] |
| Wealth Status |  |  |  |  |  |  |
| Poor | Ref | Ref | Ref | Ref | Ref | Ref |
| Middle | 1.41 [1.01,1.96]^*^ | 0.81 [0.54,1.21] | 1.40 [0.92,2.13] | 1.08 [0.68,1.70] | 1.42 [1.12,1.80]^**^ | 1.26 [0.98,1.61] |
| Rich | 3.69 [2.41,5.65]^***^ | 1.33 [0.67,2.63] | 2.01 [1.44,2.81]^***^ | 1.18 [0.59,2.38] | 1.25 [1.04,1.50]^*^ | 0.88 [0.70,1.10] |
| Residence |  |  |  |  |  |  |
| Rural | Ref | Ref | Ref | Ref | Ref | Ref |
| Urban | 2.47 [1.72,3.54]^***^ | 1.52 [0.90,2.54] | 1.73 [1.23,2.43]^**^ | 0.74 [0.37,1.51] | 1.36 [1.07,1.74]^*^ | 1.15 [0.87,1.52] |
| Media Exposure |  |  |  |  |  |  |
| Low | Ref | Ref | Ref | Ref | Ref | Ref |
| Moderate | 1.29 [0.98,1.70] | 0.85 [0.61,1.18] | 1.76 [1.28,2.42]^***^ | 1.49 [1.05,2.13]* | 1.40 [1.17,1.67]^***^ | 1.24 [1.02,1.49]* |
| High | 2.55 [1.52,4.25]^***^ | 0.63 [0.32,1.22] | 3.31 [2.05,5.34]^***^ | 2.03 [1.15,3.57]* | 1.98 [1.42,2.78]^***^ | 1.57 [1.08,2.29]* |
| Health Insurance Cover |  |  |  |  |  |  |
| No | Ref | Ref | Ref | Ref | Ref | Ref |
| Yes | 1.84 [0.45,7.60] | 0.47 [0.10,2.18] | 3.68 [1.49,9.06]^**^ | 2.52 [0.98,6.49] | 1.42 [0.57,3.53] | 0.9 [0.35,2.33] |

AOR is the adjusted odds ratio, UOR is the unadjusted odds ratio, ref is the reference; Exponentiated coefficients; 95% confidence intervals in brackets

^*^ *p* < 0.05, ^**^ *p* < 0.01, ^***^ *p* < 0.001
