# Supplementary material for: Prenatal care coverage and correlates of HIV testing in sub-Saharan Africa: Insight from demographic and health surveys of 16 countries
Source: PLoS One. 2020 Nov 9;15(11):e0242001. doi: 10.1371/journal.pone.0242001 (PMC7652338; doi:10.1371/journal.pone.0242001)
Supplement: S6 Table — (DOCX) [file pone.0242001.s006.docx]

Table S6: Adjusted and unadjusted logistic regression models showing factors associated with prenatal uptake of HIV testing in Mozambique

| Variables | Uptake of HIV testing during pregnancy | |
| --- | --- | --- |
|  | Mozambique | |
| Knowledge of MTCT | UOR [95% CI] | AOR [95% CI] |
| Low | Ref | Ref |
| Moderate | 2.82 [2.10,3.78]^***^ | 2.13 [1.56,2.91]*** |
| High | 3.30 [2.58,4.21]^***^ | 2.22 [1.70,2.88]*** |
| Age group in years |  |  |
| 15-19 | Ref | Ref |
| 20-24 | 1.09 [0.80,1.48] | 1.06 [0.76,1.49] |
| 25-34 | 0.85 [0.63,1.13] | 0.93 [0.67,1.29] |
| 35-49 | 0.58 [0.42,0.80]^**^ | 0.88 [0.61,1.27] |
| Marital Status |  |  |
| Never Married | Ref | Ref |
| Currently married | 0.52 [0.34,0.78]^**^ | 1.03 [0.64,1.64] |
| Previously married | 0.62 [0.39,1.00]^*^ | 1.1 [0.65,1.88] |
| Cohabiting | 1.05 [0.69,1.60] | 1.52 [0.95,2.46] |
| Education level |  |  |
| None | Ref | Ref |
| Primary | 2.07 [1.65,2.59]^***^ | 1.51 [1.18,1.94]** |
| Secondary & Higher | 9.81 [6.43,14.97]^***^ | 3.50 [2.14,5.71]*** |
| Wealth Status |  |  |
| Poor | Ref | Ref |
| Middle | 1.47 [1.13,1.92]^**^ | 1.3 [0.99,1.72] |
| Rich | 5.74 [4.40,7.50]^***^ | 2.80 [1.97,3.98]*** |
| Residence |  |  |
| Rural | Ref | Ref |
| Urban | 3.09 [2.41,3.96]^***^ | 1.02 [0.74,1.41] |
| Media Exposure |  |  |
| Low | Ref | Ref |
| Moderate | 1.86 [1.50,2.32]^***^ | 1.05 [0.82,1.34] |
| High | 10.15 [5.47,18.85]^***^ | 2.21 [1.11,4.39]* |
| Health Insurance Cover |  |  |
| No | Ref | Ref |
| Yes | 2.77 [0.83,9.19] | 0.63 [0.18,2.26] |

AOR is the adjusted odds ratio, UOR is the unadjusted odds ratio, ref is the reference; Exponentiated coefficients; 95% confidence intervals in brackets

^*^ *p* < 0.05, ^**^ *p* < 0.01, ^***^ *p* < 0.001
