# Supplementary material for: Prenatal care coverage and correlates of HIV testing in sub-Saharan Africa: Insight from demographic and health surveys of 16 countries
Source: PLoS One. 2020 Nov 9;15(11):e0242001. doi: 10.1371/journal.pone.0242001 (PMC7652338; doi:10.1371/journal.pone.0242001)
Supplement: S7 Table — (DOCX) [file pone.0242001.s007.docx]

Table S7: Antenatal care, HIV prevalence and new HIV infections

| Country | Women aged 15 to 49 HIV prevalence rate | Antenatal care from a skilled provider Five years preceding the survey | Children (0-14) newly infected with HIV |
| --- | --- | --- | --- |
| Central Africa |  |  |  |
| Angola | 2.7 [2.3 - 3.2] | 81.6 | 7000 |
| Cameroon | 4.7 [4.1 - 5.3] | 87 | 4500 |
| Chad | 1.5 [1.2 - 1.9] | 63.7 | 2200 |
| West Africa |  |  |  |
| Benin | 1.3 [0.8 - 2.0] | 83.2 | <500 |
| Guinea | 1.9 [1.5 - 2.2] | 80.9 | 1300 |
| Mali | 1.8 [1.4 - 2.2] | 79.5 | 3500 |
| Senegal | 0.5 [0.5 - 0.6] | 97.1 | <500 |
| East Africa |  |  |  |
| Burundi | 1.3 [1.1 - 1.6] | 99.2 | 820 |
| Ethiopia | 1.4 [1.0 - 1.8] | 62.4 | 2700 |
| Rwanda | 3.2 [2.6 - 3.6] | 97.6 | <500 |
| Uganda | 7.1 [6.6 - 7.7] | 97.3 | 7500 |
| Southern Africa |  |  |  |
| Malawi | 10.8 [9.4 - 11.7] | 94.8 | 2500 |
| Mozambique | 15.2 [12.2 - 19.0] | 87.2 | 15 000 |
| South Africa | 25.8 [22.3 - 28.6] | 93.7 | 14 000 |
| Zambia | 13.8 [12.1 - 15.4] | 96.9 | 5400 |
| Zimbabwe | 15.4 [13.0 - 17.5] | 93.3 | 4800 |

Source: The DHS Program STATcompiler and UNAIDS 2019 estimates
