# Supplementary material for: Prenatal care coverage and correlates of HIV testing in sub-Saharan Africa: Insight from demographic and health surveys of 16 countries
Source: PLoS One. 2020 Nov 9;15(11):e0242001. doi: 10.1371/journal.pone.0242001 (PMC7652338; doi:10.1371/journal.pone.0242001)
Supplement: Attachment — Submitted filename: PlosOne paper review_DHS_PMTCT_Aug2020.docx [file pone.0242001.s008.docx]

**PlosOne paper review: PONE-D-20-21800 –**

**Knowledge of mother-to-child transmission of HIV and uptake of HIV testing during pregnancy: findings from 14 sub-Saharan African countries**

**Overall comments:**

- The DHS is a cross-sectional study, which means you can’t draw directional associations, or cause-and-effect. Therefore, you cannot say that education caused testing (direction of effect), because it’s just as plausible that testing access resulted in greater knowledge of MTCT, especially since education is usually part of the pre-test process in ANC. To be able to draw the conclusion you’ve drawn, you’d have to have some indication that women had been educated on MTCT prior to their test, something hard to do in a cross-sectional study if there wasn’t a question specifically targeting when the person gained knowledge about PMTCT
- There’s no reflection in your methods, results or discussion about the possibility of collinearity and confounding variables (ie. media exposure and knowledge) – this is a gap that needs to be addressed
- Your main independent variable and your dependent variable are almost certainly highly correlated. I’d suggest that this is because education on PMTCT happens in ANC as part of the pre-test counseling process, so knowledge=testing. This is really important and isn’t addressed anywhere in your paper or accounted for in your analysis plan.
- I think your discussion needs to dig a little deeper into what your results are telling you:
  - It’s clear that media exposure is associated with testing, which implies that the knowledge influencing behavior (testing) comes from somewhere other than the ANC setting. But the analysis doesn’t account for this, and there’s no mention of this in the discussion
  - Looking at marital status and testing – the relationship is the inverse of what you’d want to see; marriage/cohabitating is associated with not testing – other national PMTCT evaluations from SSA show similar findings and it’s worth discussing this in your paper because it appears to hold true across multiple countries in your analysis
- In the intro section more work needs to be done to highlight the major variation in PMTCT success within Africa. Several countries have demonstrated successful MTCT at the levels seen in developed nations (see Zim, SA and Malawi publications), but this doesn’t come out clearly when all grouped together as ‘PMTCT programmes in SSA’.
- Media exposure classification for Zambia needs to be revised – this appears to be inconsistent with classification for all other countries – if ‘almost every day’, then this should be grouped with the high media exposure, not moderate media exposure
- It’s unclear from the description in the methods whether weighting was done correctly during analysis. Weight variables are usually provided with DHS datasets and the use of these in your analysis should be described in your methods, and your results presented as ‘weighted’
- Table content and formatting: The table selection, content and formatting needs to be worked on. Suggest reviewing other PMTCT papers with regression analyses and identifying the ‘typical’ flow of tables included in the results sections.

**Specific comments:**

Abstract

Line 34 – ‘inefficient’ isn’t a good word here. Please identify a better descriptor: ie. ‘Incomplete’? ‘Inadequate coverage of’? ‘inadequate uptake’?

Line 38 – what software was used?

Introduction

Line 64-66 – sentences 1 and 2 are repetitive. Combine into a single sentence.

Line 77 – update your literature review to include National PMTCT evaluations which have been published since 2015 from Malawi, Zimbabwe and South Africa – all have demonstrated in nationally representative studies that MTCT at 6-12wks postpartum is comparable to ‘global north’. Suggest you focus the intro section on the wide variation of PMTCT success within Africa for greater impact to the reader.

Line 80-82 – revise sentence based on guidance provided in previous comment

Line 86 – not clear what ‘operate through knowledge’ means – revise sentence

Line 94 – please provide reference(s) for first sentence in the paragraph

Line 102 – remove ‘in the main’. Suggest starting sentence with “we theorized that”

Lines 108-118 – move to methods section, and remove the sentences about how the findings can be used (those below in your conclusions section if still relevant after doing the analysis)

Methods

Line 120 – ‘uses’ should be ‘used’ – make sure you use past tense consistently throughout the paper (ie see lines 124, 125 and 126 which also contain present tenses)

Line 124 – Some of the countries excluded from the study would have met the criteria as described (ie. Malawi). Please be more specific if there were questions/data specifically needed for the analysis that excluded some countries.

Line 130 -the sentence on weighted sounds like a conclusion from the previous sentence. Weighting needs to be described briefly as it’s own step in the analysis, not as part of the study population

Line 159 – remove ‘also’.

Line 167 – why would ‘almost every day’ be moderate and not high exposure, when the other questions of ‘at least once a week’ count as high for each type of media? If the ‘3’ refers to the individual question (ie. 3 on a scale of 0-2), then that should be made clear, and then the high media category would become ≥4 rather than 4-6.

Line 172 – sample weights should be described as ‘women’s sample weights’, but just as ‘Weighting’

Line 180 – ‘pooled the data to create a single dataset’ is more accurate

Results

Line 189 – use present tense ‘present’, not ‘presented’ (sorry, a bit confusing but now that you are presenting your results in the paper, you use the present tense)

Lines 215-222 – The description of your analysis belongs in your methods section. The results section should only include results.

Line 216-217 – is Model 1 not just your unadjusted analysis presented above in Table 3?

Where is the univariable analysis that shows the association between each of the covariates and the outcome? They should be included in your Table 3, with additional columns – it’ll likely take up an entire page in landscape orientation but that’s fine.

For model 2, did you identify which covariates were significantly associated with transmission in before fitting them in the model? Or did you just include everything in the model? Need to explain how multivariates were selected

Line 220 – “Direction of effect” cannot be established from a cross-sectional study design. You cannot claim cause and effect. You can only demonstrate an association between variables.
