# Supplementary material for: Prenatal care coverage and correlates of HIV testing in sub-Saharan Africa: Insight from demographic and health surveys of 16 countries
Source: PLoS One. 2020 Nov 9;15(11):e0242001. doi: 10.1371/journal.pone.0242001 (PMC7652338; doi:10.1371/journal.pone.0242001)
Supplement: Attachment — Submitted filename: _Reponse to Reviewers PONE-D-20-21800.docx [file pone.0242001.s009.docx]

**PlosOne paper review: PONE-D-20-21800 –**

**Title of paper: Knowledge of mother-to-child transmission of HIV and uptake of HIV testing during pregnancy: findings from 14 sub-Saharan African countries**

Dear Editor:

I am pleased to re-submit the revised version of our manuscript. We appreciate the time taken to critically review this paper and the constructive comments we received from you and the anonymous reviewers. We believe that addressing all of your concerns as well as theirs, has contributed significantly to our paper. We have considered all the comments and have addressed each of the concerns as outlined below.

On behalf of all authors

***Editors comments***

Many thanks for your submission which highlights important discrepancies in and consequences of vertical transmission of HIV policies across the region. Both reviewers have highlighted concerns regarding the presentation of the methodology and the conclusions drawn. The premise that there is a straight-forward causal relationship between knowledge and testing needs to be examined in more detail to account for study design, confounding and collinearity. Here regional PMTCT policy and practice are important: education and counselling as part of PMTCT, opt-out testing versus opt-in etc. Similarly, as noted in the discussion, ANC attendance is vital.

**Response**: We thank the editor and the reviewers for all these insightful comments. We have addressed these comments, making changes to the methods, results, and addressing the issues of collinearity and regional PMTCT policy and practice. We have specifically emphasized why ANC attendance is critical to the uptake of HIV testing during pregnancy. Also, our country stratified analysis suggests that knowledge of MTCT is important for HIV testing uptake given this variable was significant for all countries included in the analysis. We have now focused on all factors associated with uptake of HIV testing during pregnancy.

**Comment**

Please check the formatting of the tables, including denominators (e.g. Table 2), units (e.g. Table 3) and general formatting (e.g. Table 5) where appropriate.

***Response***: We have made these corrections to the tables. Many thanks for suggesting these corrections.

***Reviewer 1***

***Comments***

Reviewer #1: Knowledge of mother-to-child transmission of HIV and uptake of HIV testing during pregnancy: findings from 14 sub-Saharan African countries
Awopegba OE, et al
Comments
Abstract
Line 49-51:‘The odds of prenatal care HIV testing was significantly higher [21.21, CI=20.03,22.46] in countries where most women knew of MTCT compared to countries where most women did not know of MTCT routes of transmission.’
**Comment**
While from the these results are true based on the data used, but there some confounding factors, for example in Uganda it is kind of a must for every pregnant mother who attends ANC to test for HIV, rather than a voluntary choice of the mother. So these health systems differences across the countries have to be taken into account, before we conclude that the testing rates of HIV during prenatal are due to the level of knowledge. Like I have mentioned in these countries like Rwanda and Uganda, HIV testing is ‘mandatory’ during ANC, and the mothers are also given health education. One would love to know the health systems dynamics for PMTCT in West Africa, where testing and knowledge are low, so as to put these findings into context.
Line 53-56: The conclusion of ‘educating women of the risk of MTCT could be an important strategy for increasing HIV testing uptake, especially in West and Central Africa, where the rate of testing during pregnancy remains low, and the rate of MTCT remain high, is not the only solution, when other factors are not addressed, because knowledge alone without addressing health systems barriers is not enough.

**Response**: We thank the reviewer for these comments. We have used this insight to improve our introduction and discussion. Also, we have revised the conclusion to highlight the role of other factors in our findings.

**Comment**

Line 77-78.’ Global north countries with a universal prenatal care HIV screening among pregnant women have nearly eliminated MTCT of HIV.’ Need to provide the source of this information. But also this statement underscores the importance of having prenatal care HIV screening among pregnant women. It is not clear if these services are available in West Africa, where MTCT HIV rates are high and testing is low.

**Response:** We have provided a reference to this sentence. HIV testing is generally available in all West African countries; however, the challenge remains to ensure women access prenatal care and also ensure all women who do are tested as part of prenatal care. In Nigeria, for example, not all women who received prenatal care are tested. As such, significant gaps remain in terms of universal testing of women who present for antenatal care. Nevertheless, the larger percentage of unreached women remains those who never received prenatal care.

**Comment**
Line 89-91: The authors emphasize the importance of knowledge in facilitation HIV testing among prenatal mothers, but literature has shown that knowledge alone is not enough.
‘Knowledge of MTCT is considered essential to facilitate HIV testing since women with knowledge of HIV transmission have a better understanding and appreciation of the need for HIV testing and the perceived benefit of testing not only for them but also for their infant 17’.

**Response**: We agree with this comment and have revised the manuscript accordingly. We have highlighted the role of PMTCT policies, health system strengthening, and addressing of demand and supply factors in improving coverage of prenatal care testing.

**Comment**
Line 92-107: The authors are using The Health Belief Model (HBM) with the premise that knowledge is enough for mothers to test. I feel they should have used more comprehensive models of behaviour change e.g. the approach the Capability, Opportunity and Motivation Model of Behaviour (COM-B) model (Michie S 2011), see figure below. According to the model, behaviour is a product of three necessary conditions; capability, opportunity, and motivation. Capability can be psychological (knowledge) or physical (skills), opportunity can be social (societal influences) or physical (environmental resources) while motivation can be automatic (emotion) or reflective (beliefs, intentions). Such model like COM-B gives a comprehensive analysis of the issues that affect prenatal HIV testing. Therefore, the current analysis gives a narrow picture.

Figure 1. COM-B Model Michie S 2011.

Therefore, the authors need to recognize the limitations of their theoretical approach, is not comprehensive enough in exploring the factors that affect prenatal HIV testing. But also the secondary data limitations, not able to have collected other data such as health systems factors e.g availability of testing services, how testing is done, etc.Response: We appreciate the reviewer for not only providing comments but also suggesting a theory. We have read this theory and have applied it in this study. The theory provides a comprehensive analysis of barriers and enablers of HIV testing and we have related the constructs of the theory to our study.

Line 210-2013: Table 3. The authors don’t give the reference point and also should provide the confidence intervals.

Response: Table 3 is shows the cross-tabulated results of knowledge and prenatal care testing from a chi-square test of independence. Hence, it does not require confidence intervals intervals nor reference point as required in a binary logistic regression table.

Line 2017-2025: In all countries, women who had moderate to high knowledge of MTCT had higher odds of HIV testing uptake during pregnancy compared with women who had low MTCT 219 knowledge. In Model 2, we added sociodemographic factors, media exposure, and health insurance as covariates. After adjusting for covariates, the magnitude and direction of effect persisted, indicating a strong and robust effect of MTCT knowledge on uptake of HIV testing during pregnancy. The odds of prenatal HIV testing uptake was higher among women with high MTCT 223 knowledge, especially in Chad (AOR: 38.30; 95% CI: 26.23, 55.93), Benin (AOR: 40.65; 95% CI: 224 31.95, 51.72), Angola (AOR:8.14; 95% CI:6.77, 9.78), Burundi (AOR: 53.37; 95% CI: 36.00, 225 79.11), and Zimbabwe (AOR: 19.1; 95% CI: 10.4, 35.0).
Comment: Though this data analysis is correct, it has a limitation of health systems issues on the ground.

**Response:** We have highlighted this limitation under the strengths and limitations of our study.

Table 4; Also does not provide reference points/measurements.

Comment: It would also be important for this study to give us a clue on the prevalence of couple testing, given that the pregnant mother testing alone without the partner leaves a gap in terms of achieving the PMTCT targets.

The authors should acknowledge the limitations of this secondary data analysis.

**Response:** The reference point in Table 4 is denoted by “Ref”. In relation to couple testing, we have acknowledged this as a limitation since the variable was not available in the datasets.

***Review 2***

***Comments:***

- The DHS is a cross-sectional study, which means you can’t draw directional associations, or cause-and-effect. Therefore, you cannot say that education caused testing (direction of effect), because it’s just as plausible that testing access resulted in greater knowledge of MTCT, especially since education is usually part of the pre-test process in ANC. To be able to draw the conclusion you’ve drawn, you’d have to have some indication that women had been educated on MTCT prior to their test, something hard to do in a cross-sectional study if there wasn’t a question specifically targeting when the person gained knowledge about PMTCT

**Response**: We did indicate this under our study limitation.

- There’s no reflection in your methods, results or discussion about the possibility of collinearity and confounding variables (ie. media exposure and knowledge) – this is a gap that needs to be addressed

**Response**: We have now addressed this.

- Your main independent variable and your dependent variable are almost certainly highly correlated. I’d suggest that this is because education on PMTCT happens in ANC as part of the pre-test counseling process, so knowledge=testing. This is really important and isn’t addressed anywhere in your paper or accounted for in your analysis plan.

**Response**: The correlation coefficient was approximately 0.5. We agree that this is high and have discussed it.

- I think your discussion needs to dig a little deeper into what your results are telling you:
  - It’s clear that media exposure is associated with testing, which implies that the knowledge influencing behavior (testing) comes from somewhere other than the ANC setting. But the analysis doesn’t account for this, and there’s no mention of this in the discussion
- **Response**: We have included this in our discussion.
  - Looking at marital status and testing – the relationship is the inverse of what you’d want to see; marriage/cohabitating is associated with not testing – other national PMTCT evaluations from SSA show similar findings and it’s worth discussing this in your paper because it appears to hold true across multiple countries in your analysis
- **Response**: We have discussed the effect of marital status. However, we found a significant association between marital status and tesing only in 2 of the 16 countries.
- In the intro section more work needs to be done to highlight the major variation in PMTCT success within Africa. Several countries have demonstrated successful MTCT at the levels seen in developed nations (see Zim, SA and Malawi publications), but this doesn’t come out clearly when all grouped together as ‘PMTCT programmes in SSA’.

**Response**: We have specifically referenced these countries in the introduction. We have further highlighted the remarkable progress made particularly by southern African countries, including Zim, SA and Malawi.

- Media exposure classification for Zambia needs to be revised – this appears to be inconsistent with classification for all other countries – if ‘almost every day’, then this should be grouped with the high media exposure, not moderate media exposure

**Response**: We have grouped ‘almost every day’as high media exposure.

- It’s unclear from the description in the methods whether weighting was done correctly during analysis. Weight variables are usually provided with DHS datasets and the use of these in your analysis should be described in your methods, and your results presented as ‘weighted’

**Response**: Women’s sample weights were appropriately applied to obtain unbiased estimates according to the DHS guidelines. We have made this clear in the methods.

- Table content and formatting: The table selection, content and formatting needs to be worked on. Suggest reviewing other PMTCT papers with regression analyses and identifying the ‘typical’ flow of tables included in the results sections.

**Response**: We have revised the tables and formatted them appropriately.

**Specific comments:**

Abstract

Line 34 – ‘inefficient’ isn’t a good word here. Please identify a better descriptor: ie. ‘Incomplete’? ‘Inadequate coverage of’? ‘inadequate uptake’?

**Response**: We thank the reviewer for this correction. We have effected the change.

Line 38 – what software was used?

**Response**: We used Stata version 16 and have indicated this at the statistical analyses section.

Introduction

Line 64-66 – sentences 1 and 2 are repetitive. Combine into a single sentence.

**Response**: Done

Line 77 – update your literature review to include National PMTCT evaluations which have been published since 2015 from Malawi, Zimbabwe and South Africa – all have demonstrated in nationally representative studies that MTCT at 6-12wks postpartum is comparable to ‘global north’. Suggest you focus the intro section on the wide variation of PMTCT success within Africa for greater impact to the reader.

**Response**: We thank the reviewer for the positive feedback. We have focused on introduction on the wide variation in PMTCT success within Africa

Line 80-82 – revise sentence based on guidance provided in previous comment

**Response**: We thank the reviewer for important suggestions. We have revised the sentence.

Line 86 – not clear what ‘operate through knowledge’ means – revise sentence

**Response**: deleted

Line 94 – please provide reference(s) for first sentence in the paragraph

**Response**: Done

Line 102 – remove ‘in the main’. Suggest starting sentence with “we theorized that”

**Response**: We have deleted the sentence

Lines 108-118 – move to methods section, and remove the sentences about how the findings can be used (those below in your conclusions section if still relevant after doing the analysis)

**Response**: done

Methods

Line 120 – ‘uses’ should be ‘used’ – make sure you use past tense consistently throughout the paper (ie see lines 124, 125 and 126 which also contain present tenses)

**Response**: We have changed to past tense.

Line 124 – Some of the countries excluded from the study would have met the criteria as described (ie. Malawi). Please be more specific if there were questions/data specifically needed for the analysis that excluded some countries.

**Response**: We have added two more countries(Malawi and Mozambique).

Line 130 -the sentence on weighted sounds like a conclusion from the previous sentence. Weighting needs to be described briefly as it’s own step in the analysis, not as part of the study population

**Response**: We described weighting under data analysis. Here we only indicated the weighted sample included in the study as shown in Table 1.

Line 159 – remove ‘also’.

**Response**: Done

Line 167 – why would ‘almost every day’ be moderate and not high exposure, when the other questions of ‘at least once a week’ count as high for each type of media? If the ‘3’ refers to the individual question (ie. 3 on a scale of 0-2), then that should be made clear, and then the high media category would become ≥4 rather than 4-6.

**Response**: We have revised this accordingly.

Line 172 – sample weights should be described as ‘women’s sample weights’, but just as ‘Weighting’

**Response**: We have revised accordingly

Line 180 – ‘pooled the data to create a single dataset’ is more accurate

**Response**: We have revised the sentence accordingly.

Results

Line 189 – use present tense ‘present’, not ‘presented’ (sorry, a bit confusing but now that you are presenting your results in the paper, you use the present tense)

**Response**: Corrected

Lines 215-222 – The description of your analysis belongs in your methods section. The results section should only include results.

**Response**: We have deleted the sentences belonging to the methods.

Line 216-217 – is Model 1 not just your unadjusted analysis presented above in Table 3?

**Response**: Table 3 is a descriptive table. The p-values are from Pearson chi-square.

Where is the univariable analysis that shows the association between each of the covariates and the outcome? They should be included in your Table 3, with additional columns – it’ll likely take up an entire page in landscape orientation but that’s fine.

**Response**: We have revised table 3. It now shows association between the covariates and the outcomes.

For model 2, did you identify which covariates were significantly associated with transmission in before fitting them in the model? Or did you just include everything in the model? Need to explain how multivariates were selected

**Response**: We included all these variables based on previous studies indicating they were significantly associated with testing.

Line 220 – “Direction of effect” cannot be established from a cross-sectional study design. You cannot claim cause and effect. You can only demonstrate an association between variables.

**Response**: We have revised accordingly.
